# Canine and feline papillomaviruses: an update

**DOI:** 10.3389/fvets.2023.1174673

**Published:** 2023-05-16

**Authors:** Beatriz Medeiros-Fonseca, Ana I. Faustino-Rocha, Rui Medeiros, Paula A. Oliveira, Rui M. Gil da Costa

**Affiliations:** ^1^Institute for Innovation, Capacity Building and Sustainability of Agri-food Production (Inov4Agro), Centre for Research and Technology of Agro-Environmental and Biological Sciences (CITAB), Vila Real, Portugal; ^2^Molecular Oncology and Viral Pathology Group, Research Center of IPO Porto (CI-IPOP)/Health Research Network (RISE)@CI-IPOP, Portuguese Oncology Institute of Porto (IPO Porto)/Porto Comprehensive Cancer Center (Porto.CCC), Porto, Portugal; ^3^Department of Zootechnics, School of Sciences and Technology, University of Évora, Évora, Portugal; ^4^Comprehensive Health Research Center (CHRC), Évora, Portugal; ^5^Faculty of Medicine, University of Porto, Porto, Portugal; ^6^Abel Salazar Institute for the Biomedical Sciences, Porto, Portugal; ^7^FP-I3ID, FP-ENAS, FP-BHS, University Fernando Pessoa, Porto, Portugal; ^8^Faculty of Health Sciences, University Fernando Pessoa, Porto, Portugal; ^9^Research Department, Portuguese League Against Cancer (NRNorte), Porto, Portugal; ^10^Department of Veterinary Sciences, University of Trás-os-Montes and Alto Douro, Vila Real, Portugal; ^11^Laboratory for Process Engineering, Environment, Biotechnology and Energy (LEPABE), Faculty of Engineering, University of Porto, Porto, Portugal; ^12^Postgraduate Programme in Adult Health (PPGSAD), Department of Morphology, Federal University of Maranhão (UFMA), UFMA University Hospital (HUUFMA), São Luís, Brazil

**Keywords:** cancer, cat, dog, human, papillomavirus

## Abstract

Papillomaviruses are small viruses able to cause disease not only in mammalians, but also in birds and reptiles. In recent years, a rising number of papillomaviruses have been identified in dogs and cats, totaling 24 canine papillomavirus (CPV) and six feline papillomavirus (FcaPV). The canine and feline papillomaviruses (CPVs and FcaPVs, respectively) are responsible for multiple lesions in these domestic species but the potential pathological relevance of some recently identified types remains to be determined. CPVs are associated with oral papillomatosis, cutaneous papillomas and viral pigmented plaques, and have been rarely associated with the development of oral and cutaneous squamous cell carcinomas in their canine hosts. FcaPVs are associated with oral papillomas, viral plaques, and Bowenoid *in situ* carcinomas. The present review provides readers with the more recent advances on dog and cat papillomavirus research, bringing an update on this field to both veterinary practitioners and the virology community at large.

## 1. Introduction

Papillomaviruses are small, icosahedral, non-enveloped viruses with a capsid that involves the genome of a double-stranded circular DNA molecule. These viruses are able to cause multiple epithelial lesions not only in mammalians, but also in birds and reptiles (1). Their genome contains approximately 8,000 base pairs (bp) and includes five or six early (E) and two late (L) open reading frames (ORF) (2). Papillomaviruses are classified considering the L1 ORF sequence, with the papillomavirus of the same genus having more than 60% L1 ORF similarity and presenting similar host, location, and behavior. Different types of papillomaviruses have a similarity lower than 90% in the L1 ORF sequence (3). Additionally to this genome-based taxonomic classification, papillomaviruses may be also classified as those that cause hyperplastic papillomas (warts) or those that infect without causing clinical lesions (asymptomatic) (4). The papillomaviruses that cause warts may be transmitted between animals directly or via fomites, and mainly affect young adults. After infection, these papillomaviruses stimulate fast replication of epithelial cells, leading to epithelial hyperplasia and the development of a wart (5–7). In turn, warts stimulate an immune response inhibiting virus replication that results in the spontaneous resolution of the lesion (8). The majority of papillomaviruses do not cause a visible lesion, because they stimulate slow replication of epithelium (9, 10), and, usually, they are acquired during or soon after birth (11). Although there are few studies addressing the subclinical infection by papillomavirus in dogs and cats, a subclinical infection was already noticed in these species, suggesting that it is ubiquitous in companion animals, as observed in humans (12–16). Furthermore to the classifications presented above, as some of the papillomaviruses can be only detected in hyperplastic lesions and others may be detected in neoplastic lesions, the papillomavirues may also be classified as low-risk types (those asymptomatic or causing self-resolving warts) or high-risk types (those can cause neoplasia) (6).

Excepting some types of papillomaviruses from genus *Deltapapillomavirus*, papillomaviruses are highly species-specific (3, 6, 12). Bearing in mind their tissue specificity, papillomaviruses can be classed into those that affect cutaneous sites and those affecting mucosal sites. The life cycle of papillomavirus is coordinated with the division and maturation of stratified epithelium cells (4, 17). A papillomavirus infection initiates with the contact of the viral particles with the basal cells of the epithelium, after microtrauma (18). After infecting the keratinocytes of the basal layer, the viral genome multiples within the suprabasal epithelial layers. As the cells replicate, the daughter cells contain the papillomavirus DNA, allowing the persistence of the infection. The continuous replication of epithelial cells infected with the virus allows viral genome amplification leading to the formation of cells containing multiple viral copies (19).

Papillomaviruses induce a wide spectrum of lesions in animals, ranging from short-lived papillomas that regress spontaneously, to cancers. This behavior can be observed with those papillomaviruses infecting companion animals—the canine papillomaviruses (CPVs) and feline papillomaviruses (FcaPVs), with an increasing number of new viruses infecting dogs and cats identified over the years. There are many similarities between the diseases caused by papillomaviruses in humans and those caused by these viruses in animals like cattle, dogs, and cats (20). Considering the species and tissue specificity of papillomaviruses, their study in animals is essential to better understand viral biology and pathogenesis, and to search for new and more effective strategies to fight viral infection and its consequences (21, 22). Accordingly, this work aims to provide the readers with the more recent advances on dog and cat papillomavirus research, bringing an update on this field to both veterinary practitioners and scientific community.

## 2. Papillomaviruses in animals

Approximately 450 different types of human papillomavirus (HPV), classed into five genera (*Alphapapillomavirus*, *Betapapillomavirus*, *Gammapapillomavirus*, *Mupapillomavirus*, and *Nupapillomavirus*), were identified over the years (4). A lower number of papillomaviruses were identified in animals (23). However, the increasing interest and study of animal papillomaviruses in the last years, allowed the identification of more non-human papillomaviruses. Some of the best known papillomaviruses among animals affect mostly domestic species, since they are closer to humans and their lesions are more frequently detected by owners and reported (20). Ongoing studies in this field will continue to unveil more human and non-human papillomavirus and viral types.

## 3. Canine papillomaviruses (CPVs)

Presently, 24 types of CPVs have been identified in dogs, most of which associated with both mucosal and cutaneous lesions. Most of the types belong to the genus *Chipapillomavirus* (CPV 3, 4, 5, 8, 9, 10, 11, 12, 14, 15, 16, 18, 19, and 24), and the remaining types belong to the genus *Lambdapapillomavirus* (CPV 1 and 6) or the genus *Taupapillomavirus* (CPV 2, 7, 13, 17, 20, 21, 22, and 23) (Table 1). These papillomaviruses have tropism for different organs, with almost of all affecting the skin. Some of them, namely CPVs 1, 2, 3, 4, 6, 8, 13, 17, and 19, have tropism for more than one organ, affecting the skin and oral cavity. However, the tissue tropism and the possible pathogenicity of CPVs 20, 21, 22, and 23 remains to be determined (24–26) (Table 1). Over the years, CPVs have been classically associated with oral papilloma, cutaneous papilloma, inverted papilloma, and pigmented plaques in dogs. Rarely have these viruses been associated with the development of oral and cutaneous squamous cell carcinomas (SCCs) in this species, mostly under conditions of immune suppression The *Lambdapapillomavirus* are associated with oral and cutaneous papillomas. CPV1, in particular, is also associated with inverted papillomas and conjunctival epithelial hyperplasia. The papillomavirus belonging to the Chi genus are related to viral pigmented plaques and cutaneous squamous cell carcinomas, while the *Taupapillomavirus* are linked to cutaneous papillomas (24–26) (Table 1).

**Table 1:** CPV types and their associated lesions at multiple anatomic sites.

CPV type	Genus	Anatomical distribution	Lesions	Size (bp)	Genes	References
CPV-1	*Lambdapapillomavirus*	Skin Oral cavity	Oral papillomas Cutaneous papillomas Inverted papillomas Conjunctival epithelial hyperplasia	8,607	E1, E2, E4, E6, E7, L1, L2	(24–26)
CPV-2	*Taupapillomavirus*	Skin Oral cavity	Cutaneous papillomas Oral SCC	8,101	E1, E2, E4, E5, E6, E7, L1, L2	(49)
CPV-3	*Chipapillomavirus*	Skin Oral cavity	Viral pigmented plaques Cutaneous SCC	7,801	E1, E2, E6, E7, L1, L2	(50)
CPV-4	*Chipapillomavirus*	Skin Oral cavity	Viral pigmented plaques Cutaneous SCC	7,742	E1, E2, E6, E7, L1, L2	(35)
CPV-5	*Chipapillomavirus*	Skin	Viral pigmented plaques Cutaneous SCC	7,810	E1, E2, E4, E6, E7, L1, L2	(26)
CPV-6	*Lambdapapillomavirus*	Skin Oral cavity	Oral papillomas Cutaneous papillomas	8,242	E1, E2, E4, E6, E7, L1, L2	(26)
CPV-7	*Taupapillomavirus*	Skin	Cutaneous papillomas Oral SCC	7,955	E1, E2, E4, E6, E7, L1, L2	(26)
CPV-8	*Chipapillomavirus*	Skin Oral cavity	Viral pigmented plaques Cutaneous SCC	7,784	E1, E2, E4, E6, E7, L1, L2	(15)
CPV-9	*Chipapillomavirus*	Skin	Cutaneous papillomas Viral pigmented plaques Cutaneous SCC	7,873	E1, E2, E4, E6, E7, L1, L2	(49)
CPV-10	*Chipapillomavirus*	Skin	Viral pigmented plaques Cutaneous SCC	7,774	E1, E2, E4, E6, E7, L1, L2	(51)
CPV-11	*Chipapillomavirus*	Skin	Viral pigmented plaques Cutaneous SCC	7,828	E1, E2, E4, E5, E6, E7, L1, L2	(52)
CPV-12	*Chipapillomavirus*	Skin	Cutaneous papillomas Viral pigmented plaques Cutaneous SCC	7,890	E1, E2, E4, E6, E7, L1, L2	(53)
CPV-13	*Taupapillomavirus*	Skin Oral cavity	Cutaneous papillomas Oral SCC	8,228	E1, E2, E4, E6, E7, L1, L2	(54)
CPV-14	*Chipapillomavirus*	Skin	Viral pigmented plaques Cutaneous SCC	7,826	E1, E2, E4, E6, E7, L1, L2	(55)
CPV-15	*Chipapillomavirus*	Skin	Viral pigmented plaques Cutaneous SCC	7,776	E1, E2, E6, E7, L1, L2	(28)
CPV-16	*Chipapillomavirus*	Skin	Viral pigmented plaques Cutaneous SCC	7,796	E1, E2, E4, E6, E7, L1, L2	(38)
CPV-17	*Taupapillomavirus*	Skin Oral cavity	Cutaneous papillomas Oral SCC	8,007	E2, E4, E5, E6, E7, L1, L2	(56)
CPV-18	*Chipapillomavirus*	Skin	Viral pigmented plaques	7,810	E1, E2, E4, E6, E7, L1, L2	(57)
CPV-19	*Chipapillomavirus*	Skin Oral cavity	Cutaneous papillomas Oral SCC	7,941	E1, E2, E4, E5, E6, E7, L1, L2	(58)
CPV-20	*Taupapillomavirus*	Unknown	Unknown	7,839	E1, E2, E4, E5, E6, E7, L1, L2	Unpublished
CPV-21	*Taupapillomavirus*	Detected in nasal swabs	Unknown	8,225	E1, E2, E4, E6, E7, L1, L2	(5)
CPV-22	*Taupapillomavirus*	Detected in nasal swabs	Unknown	8,300	E1, E2, E4, E6, E7, L1, L2	(5)
CPV-23	*Taupapillomavirus*	Detected in nasal swabs	Unknown	8,140	E1, E2, E4, E6, E7, L1, L2	(5)
CPV-24	*Chipapillomavirus*	Skin	Viral pigmented plaques	7,742	E1, E2, E4, E6, E7, L1, L2	(59)

Cutaneous warts have been considered the second most frequent skin tumor in dogs under 1 year of age (27). These lesions may be induced by CPV1 or CPV2, or both types simultaneously (28, 29). Warts are frequently found on the feet and around the face and ears (30). Most canine cutaneous warts regress spontaneously, within 3 months, and do not cause discomfort. However, some of them may persist for 2 years before regressing (30). The progression of cutaneous warts into SCCs is extremely rare. Cutaneous warts in the anogenital region are rarely reported in dogs (31).

Oral papillomatosis in dogs presents as multiple exophytic smooth or cauliflower-like warts on the lips and mouth. Like cutaneous papillomatosis, oral papillomas are frequent in young animals (30). Most of oral warts are caused by CPV1 and regress spontaneously within 4–8 weeks (32, 33). In few cases, dogs can develop further warts that increase in size over a year and can spread from the oral cavity to the haired skin or progress to SCC (30). Few reports have suggested that the transmission between dogs is possible (8, 34).

Viral cutaneous plaques, also called pigmented plaques, are rarely reported in dogs. The development of these lesions has been associated with *Chipapillomavirus* types, and with immunosuppressive conditions and breed predisposition (35). The pigmented plaques are usually dark and multiple, and common on the ventral and medial aspects of the limbs (36). Extensive plaque can cause pruritus and pain, but in the majority of the cases the plaques do not impact the animals’ life and can regress spontaneously (35, 36). There are reports of HPV-associated canine cutaneous and oral SCC, but there is limited evidence suggesting the involvement of papillomaviruses in this kind of lesion (37). CPV types 2–17 and CPV type 19 have been found in cutaneous and oral SCC (Table 1). However, presence of the virus is insufficient to establish its etiological role and additional molecular evidence exists in some cases to support its causal involvement in SCC (38, 39). The CPV E5, E6, and E7 proteins share some characteristics with homologous oncoproteins from HPV 16 which are involved in malignancy (40) suggesting they may, at least partially, contribute for cell transformation in similar ways (1). Each protein has different contributions in different contexts: for instance, E5 is a major transforming protein in bovine delta PVs but seems to play a less central role in HPV (41). The particular role of each gene and each CPV genotype remains to be determined. From the data summarized in Table 1, it is clear that all genotypes possess the E6 and E7 genes, regardless of whether they were found in SCC or not. Conversely, the E5 is occasionally present in both groups. It would be interesting to systematically compare the genomes of CPV genotypes suggested to be involved in SCC with those involved only in benign lesions, to identify molecular determinants of malignant transformation.

## 4. Feline papillomaviruses (FcaPVs)

Feline papillomaviruses (FcaPVs) are thought to cause oral papilloma, cutaneous papilloma, viral plaques and Bowenoid *in situ* carcinomas (BISC). There is increasing evidence that FcaPVs may also be associated with the development of cutaneous squamous cell carcinomas (SCC). So far, six types of FcaPVs were described and associated with mucosal and cutaneous lesions (Table 2), as described for the dog. These viruses were grouped into sp. genera: *Dyothetapapillomavirus*, *Lambdapapillomavirus*, and *Taupapillomaviru*. FcaPV1 belongs to the genus *Lambdapapillomavirus*, has tropism for skin and oral cavity, and is associated with the development of cutaneous and oral papillomas. FcaPV2 is part of the genus *Dyothetapapillomavirus*, has tropism for the skin and is responsible for the development of viral plaques, BISC, cutaneous SCC and basal cell carcinoma. FcaPVs 3, 4, 5, and 6 belong to the genus *Taupapillomavirus* and have tropism for different organs. FcaPVs 3 and 5 have tropism for skin, and both are associated with the development of viral plaques and BISC. Moreover, FcaPV3 is also associated with the development of skin neoplasia and cutaneous SCC. FcaPV4 has tropism for the oral cavity and is related with the development of stomatitis and BISC. FcaPV6 was detected in the nasal planum and was associated with the development of cutaneous SCC. Additionally to these viruses, cats may also be affected by bovine papillomavirus (BPV)-14, a *Deltapapillomavirus*, that is responsible for the development of feline sarcoids, as observed in other species infected with *Deltapapillomavirus* BPVs (42, 43) (Table 2).

**Table 2:** FcaPV types and their associated lesions at multiple anatomic sites. Bowenoid in situ carcinomas (BISC).

FcaPV type	Genus	Anatomical distribution	Lesions	Size (bp)	Genes	References
FcaPV1	*Lambdapapillomavirus*	Skin Oral cavity	Cutaneous Papillomas Oral papillomas	8,300	E1, E2, E4, E6, E7, L1, L2	(60)
FcaPV2	*Dyothetapapillomavirus*	Skin (epidermis and follicular infundibulum)	Viral plaques BISC Cutaneous SCC Basal cell carcinoma	7,899	E1, E2, E6, E7, L1, L2	(26)
FcaPV3	*Taupapillomavirus*	Skin (epidermis and hair follicles)	Viral plaques BISC Skin neoplasia Cutaneous SCC	7,583	E1, E2, E4, E5, E6, E7, L1, L2	(42, 43)
FcaPV4	*Taupapillomavirus*	Oral cavity	Stomatitis BISC	7,616	E1, E2, E4, E6, E7, L1, L2	(61)
FcaPV5	*Taupapillomavirus*	Skin	Viral plaques BISC	7,600	E1, E2, E4, E6, E7, L1, L2	(62)
FcaPV6	*Taupapillomavirus*	Nasal planum	Cutaneous SCC	7,453	E1, E2, E4, E6, E7, L1, L2	(60, 63)

Warts are less frequent in cats than in dogs. To date, only three cases of cutaneous warts were reported in cats, and they were small and solitary, two of them were found on the nasal planum and one on the eyelid, as previously reviewed (44). Cutaneous warts in the anogenital region were never reported in cats. Oral warts were rarely reported in cats and resolve spontaneously (45). Viral cutaneous plaques are rare in cats and affect mainly middle-aged or older animals. When compared with other breeds, Sphinx or Devon Rex cats develop plaques more frequently and at a younger age (46). These plaques present as multiple pigmented or non-pigmented lesions, that do not cause pain or pruritus, on the head, face, and neck (47).

There are strong evidence suggesting that papillomaviruses are part of the etiology of skin cancers in cats (45). FcaPV2, 3 and 6 have been associated with cutaneous SCC (Table 2). However, it is not clear whether SCC develops from viral plaques or directly from normal skin (21). As observed for canine PVs, all FcaPV genotypes contain the essential E6 and E7 genes, but only FcaPV3 has an E5 gene. Apart from some members of the *Deltapapillomavirus* genus, papillomaviruses have strong tropism for keratinizing epithelia and a recent study indicates that papillomaviruses do not frequently infect the lung, mammary gland, or the bladder of dogs and cats and, consequently, are unlikely to be determining factors for cancer development in those tissues (48).

## 5. Conclusion

To date, 24 types of CPVs and six types of FcaPV have been described, most often with tropism for the skin and oral cavity. The infection of cats by a bovine papillomavirus (BPV-14) was already reported and leads to sarcoids as in other animal species. Papillomaviruses are widely recognized as a cause of several oral and cutaneous lesions in both dogs and cats, with most of lesions are self-resolving. Papillomaviruses are also potentially related to malignant diseases in these species, especially in cats. Further research in this field will likely add new papillomavirus types to those already known and will add to our current knowledge of their epidemiology and pathologic features as well as support the development of new and more effective preventive and therapeutic approaches.

## Author contributions

All authors listed have made a substantial, direct, and intellectual contribution to the work and approved it for publication.

## Funding

The present work was supported by European Investment Funds by FEDER/COMPETE/POCI-Operational Competitiveness and Internationalization Program, and National Funds by Portuguese Foundation for Science and Technology (FCT), under the projects PI86-CI-IPOP-66-2017 (CI-IPOP), UID/AGR/04033/2020 (CITAB), LA/P/0126/2020 (Inov4Agro), UIDB/00511/2020 (LEPABE), NORTE-01-0145-FEDER-000054 (Project 2SMART), and the PhD grant no. 2020.07675.BD to BM-F.

## Conflict of interest

The authors declare that the research was conducted in the absence of any commercial or financial relationships that could be construed as a potential conflict of interest.

## Publisher’s note

All claims expressed in this article are solely those of the authors and do not necessarily represent those of their affiliated organizations, or those of the publisher, the editors and the reviewers. Any product that may be evaluated in this article, or claim that may be made by its manufacturer, is not guaranteed or endorsed by the publisher.
